# Population Status and Habitat Preferences of the Critically Endangered African White‐Backed Vulture (*Gyps africanus*) in Southwest Ethiopia Region

**DOI:** 10.1002/ece3.73730

**Published:** 2026-07-01

**Authors:** Asrat Aero Mamo, Tsegaye Gadisa, Tadesse Habtamu

**Affiliations:** ^1^ Department of Biology, College of Natural Sciences Jimma University Jimma Ethiopia

**Keywords:** African white‐backed vulture, afromontane forests, age structure, habitat preferences, population status

## Abstract

The African white‐backed vulture ( *Gyps africanus* ), a critically endangered scavenger, is rapidly declining in Africa due to habitat loss, poisoning and human activities. Understanding of population status and habitat use is essential for targeted conservation in Africa. This study examines population status and habitat preferences in Afromontane forests of southwest Ethiopia. Systematic field surveys from June 2024 to February 2025 across three habitats (abattoirs, waste disposal and breeding sites) documented population parameters and habitat preferences. A total of 1150 white‐backed vultures were observed. Sightings peaked at breeding sites during the wet season. A total of 341 individuals were counted in the abattoirs sites (mean ± SD: 3.31 ± 3.95), 262 in dump sites (mean ± SD: 3.54 ± 4.34) and 547 in breeding sites (mean ± SD: 15.19 ± 5.05), with numbers varying by daily activity and seasons. The age structure shows that adults comprised 79.5% of sightings, indicating low juvenile numbers and possible recruitment issues. These vultures showed a strong preference for roosting and nesting areas in *Juniperus procera, Olea welwetschia,* and *Podocarpus falcatus,* which were the most heavily used, highlighting the importance of Afromontane Forest. Key threats identified in the region include habitat degradation, agricultural expansion near breeding cliffs, and food competition with domestic dogs, other raptors, and wild animals. Directly protect key tree species to preserve roosting and breeding areas; manage waste at abattoirs and dumping sites to ensure stable food supplies; and conduct seasonal monitoring to maximize intervention efficiency.

## Graphical abstract

The critically endangered African white‐backed Vulture is declining rapidly across Africa due to anthropogenic threats. This study, conducted in southwest Ethiopia, quantifies its population status and reveals key habitat preferences across breeding and foraging sites. The findings provide essential data for crafting targeted conservation strategies to protect this vital scavenger.

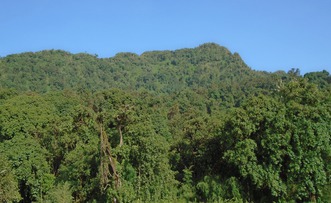

## 1 Introduction

The African White‐backed Vulture ( *Gyps africanus* ) is an Old World vulture belonging to the family Accipitridae, which also includes eagles, kites, buzzards, and hawks (Ogada et al. 2015; Mundy 2021). As one of seven vulture species found in Ethiopia, it is a resident bird often associated with human settlements, slaughterhouses and garbage dump sites (Shiferaw et al. 2022; Agunbiade et al. 2025). While it is considered the most widespread and common vulture species across Sub‐Saharan Africa, ranging from Senegal and Mali eastward to Ethiopia and Somalia, and south to South Africa, it has suffered catastrophic population declines (IUCN 2021). Consequently, *G. africanus* is currently listed as Critically Endangered on the IUCN Red List (IUCN 2021; Murn et al. 2017). Its population has declined significantly over the past three generations due to environmental and anthropogenic threats, including poisoning, habitat loss and food shortages. Historically, this species inhabits open woodlands, savannas and grasslands, primarily relying on large, scattered trees for roosting and breeding (Gandaho et al. 2025; Murn et al. 2016).

The decline of vulture population is due to a combination of human and ecological factors. Habitat destruction from agriculture, logging, and urbanization has reduced suitable roosting and nesting sites (Ogada et al. 2015; Virani et al. 2011). The species is especially vulnerable to poisoning, often from carcasses laced to target other animals (Buechley, Ruffo, et al. 2022; Ogada 2010; Swan et al. 2006). Their social feeding and wide foraging range increase the risk of mass poisoning (Phipps, Wolter, et al. 2013; Phipps, Willis, et al. 2013). Poisoning, intentional or accidental, is now a major threat, with vultures exposed to toxins in bait for large carnivores. Such practices, especially in areas of human‐wildlife conflict, have caused substantial vulture mortality (Green et al. 2004). Vultures are also ecologically important in human‐dominated areas, scavenging at slaughterhouses and dumps (Garbett et al. 2018; Kendall and Bracebridge 2021). However, rapid human population growth is the primary driver of vulture declines in sub‐Saharan Africa. This pressure disrupts landscape functioning and diminishes the ability of ecosystems to support native wildlife, including vultures and their prey (Murn and Anderson 2008). As a result, most vulture population declines stem from human activities and their adverse interactions with the species (Ogada et al. 2012).

Ethiopia is a stronghold for vultures in the Horn of Africa, including the African white‐backed vulture (Buechley, Girardello, et al. 2022). The country's diverse topography, ranging from the highlands to the Rift Valley, supports a variety of resident and migratory vulture species (Gedeon et al. 2017). However, there are few comprehensive studies on this vulture populations and threats in Southwest Ethiopia. This knowledge gap is crucial given rapid agricultural development and increasing reports of poisoning. The Kafa and Benchi Sheko zones in Southwest Ethiopia are important for studying this species due to their varied landscapes of savannas, forests, and farmland (Yilma and Yitay 2024). Despite this, little is known about the population status and habitat preferences of African white‐backed vultures in the region. The main aim of this study is to assess the current population, seasonal distribution in the study sites, and habitat preference of the African white‐backed vulture in Southwest Ethiopia.

## 2 Materials and Methods

### 2.1 Study Area

The Southwest Ethiopia Region is located in the southwestern part of the country and is home to diverse landscapes, including cloud Afromontane forests, riparian forests, and agricultural areas (Yilma and Yitay 2024; Hundera et al. 2012). This ecological diversity is further shaped by the region's climate, which experiences a uni‐modal rainfall pattern, with peak rainfall occurring between June and September (Alhamshry et al. 2020). It is classified as a warm to cool, semi‐humid zone, characterized by temperate highlands ranging from 1500 to 2500 m above sea level. The average annual temperature in the region is between 16°C and 20°C, with annual rainfall averaging around 1200 mm, reaching up to 2400 mm in the southwestern areas (Lakew and Moog 2016). The total area of the region is 39,400 km^2^.

**FIGURE 1:**
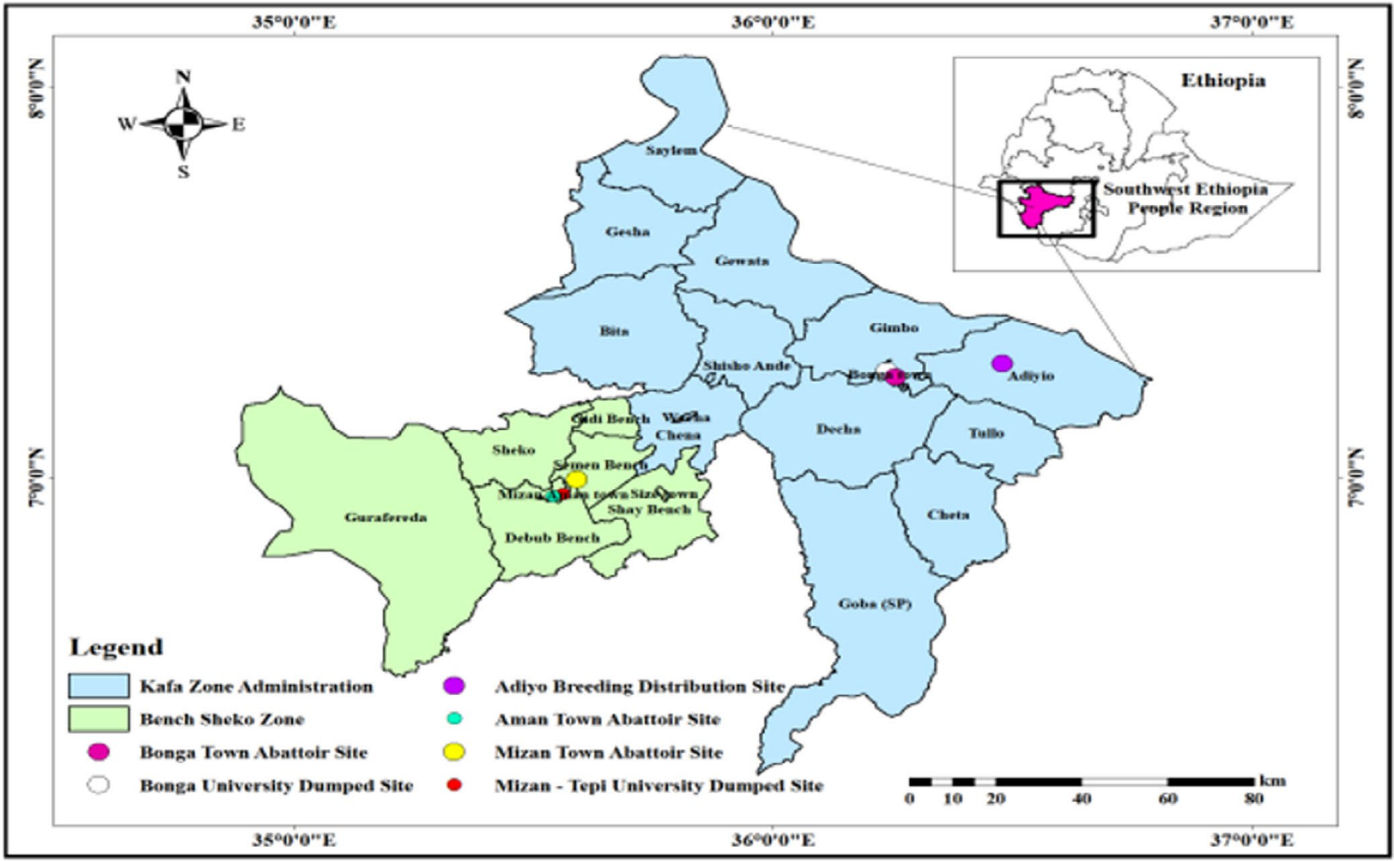
Map of study area in southwest Ethiopia.

This study was focused on selected sites within the Kafa and Benchi Sheko Zones, located at the coordinates 7°10′46.78′′N, 36°02′52.44′′E for Kafa and 6°14′60.00′′N, 35°09′60.00′′E for Benchi Sheko (Figure 1). These two zones were chosen for their ecological significance and the presence of vulture populations. The specific study sites within Kafa Zone include the Bonga abattoir site, Bonga University waste dumped site, and Adiyo district breeding site. In Benchi Sheko Zone, the study focuses on the Mizan town abattoir site, Aman town abattoir site, and Mizan‐Tepi University waste dumped site. These six sites were selected due to their high likelihood of vulture activity, particularly around the abattoirs, waste dump sites, and breeding area.

### 2.2 Study Design

The study was conducted over two distinct seasons: the dry season (December to February) and the wet season (June to August) from 2024 to 2025. Surveys were performed monthly to account for seasonal variations. Before the formal census, preliminary observations were made at all sites to understand vulture behavior at feeding (abattoirs, dump sites) and breeding areas, including activities such as feeding, roosting, and nesting.

Six study sites were selected; one breeding site (Adiyo district, the only location with confirmed nesting), three abattoirs (Bonga, Mizan, and Aman), and two dumping sites (Bonga University and Mizan‐Tepi University). Fixed‐radius point counts were established at each site. At the breeding site, a single point count station was placed at the geographical centre of the known nesting colony with a radius of 500 m, covering all active nests and adjacent perching trees. At each dumping and abattoir site, two point count stations were placed 200 m apart along the downwind edge of the facility, each with a 200 m radius. These radii were chosen after pilot observations confirmed that all visible vultures could be reliably counted without double‐counting. The total observation area was approximately 0.78 km^2^ for the breeding site and 0.25 km^2^ per anthropogenic site. This design was considered representative because additional pilot stations did not record new individuals, and the stations covered all major habitat features (open dumping ground, carcass piles, nearby perching trees).

All sites were surveyed within the same 3‐day period each month. Counts were conducted by six trained observers, each assigned to a specific site but rotated monthly to reduce observer bias. All observers received training in vulture identification and age classification before the study.

### 2.3 Data Collection

The census focused on abattoirs, waste sites and breeding areas where vultures congregated (Pomeroy et al. 2015). On each sampling day, surveys were conducted three times during peak activity windows: morning (07:00–12:00), early afternoon (12:00–16:00), and late afternoon (16:00–18:00). These windows were chosen to monitor vultures as they departed from roosts, soared on thermals, fed at abattoirs, scanned for carcasses, and returned to roosts (Thiollay 2007; Thompson et al. 2021). This schedule was maintained over three consecutive weeks each month from June 2024 to February 2025, covering both seasons.

During each session, the two observers at a site stood at the station centre and scanned the entire radius using Vortex Optics Triumph HD 10 × 42 binoculars and a Minolta Pro Shot 20MP HD camera with 67× zoom. Counts lasted 20 min per station. The maximum number of individuals of each age class seen simultaneously was recorded to avoid double‐counting. Simultaneous counts were performed by observers at different vantage points and a 3–5 min settling period was allowed after any disturbance. No significant disturbances (e.g., loud noises, approaching vehicles) occurred during counts (Gregory et al. 2004; Monadjem et al. 2012).

Vultures were identified as African White‐backed Vultures ( *Gyps africanus* ) using plumage, body size, and standard field guides. Adults were distinguished by their characteristic white back, dark wings, white neck ruff, and white underwing coverts. Juveniles were identified by their overall dark brown, streaked plumage, lacking a white back, white neck ruff, and white underwing coverts. Sex was not determined due to a lack of external dimorphism and was excluded from analyses.

For each observation, the following data were recorded: date, time of day, GPS coordinates, altitude, site type (breeding, abattoir, dumping), total vulture count, number of adults and juveniles, tree species used for perching or roosting (when applicable), and proximity to settlements. Breeding status was confirmed only at the Adiyo district site; no active nesting was found in the other zones.

### 2.4 Data Analysis

Collected data was structured and analyzed. Total abundance is the sum of all individuals counted per species across surveys. Mean abundance was calculated as the total count per species divided by number of surveys, reported as mean ± SE. A Chi‐Square Test was used to assess whether vultures showed preference for certain habitat types like tree species or site categories by comparing observed vultures in each habitat to what would be expected if they used habitats in proportion to availability. A significant result indicates preference or avoidance. Using R Studio and packages, vulture sighting locations were overlaid with habitat maps to visually and statistically correlate abundance and behavior with habitat features (Team RC 2021).

## 3 Results

### 3.1 Population Status

A total of 1150 African White‐backed vultures were recorded across 216 observations during the study period. The overall mean count per observation was 5.32 (± 6.15 SD), with counts ranging from 0 to 26 individuals. Among these, breeding sites harbored the highest number with 547 individuals (47.6%), followed by abattoir sites with 341 individuals (29.7%) and dumping sites with 262 individuals (22.8%). The mean (± SD) count per observation was markedly higher at breeding sites (15.19 ± 5.05, median = 14.0, range 4–26) compared to dumping sites (3.54 ± 4.34, median = 2.5, range 0–24) and abattoir sites (3.22 ± 3.95, median = 3.0, range 0–23) (Table 1). Tukey HSD post hoc tests confirmed that breeding sites differed significantly from both dumping (*p* < 0.0001) and abattoir sites (p < 0.0001), whereas dumping and abattoir sites did not differ (*p* = 0.872).

**TABLE 1:** Total number of white‐backed vultures counted in study sites across both seasons.

Site	Season	*n*	Total	Mean	SD	SE	Median	Min	Max	*p*
Breeding site	Wet	18	285	15.833333	5.762455	1.358223	17.0	4	26	0.45607
	Dry	18	262	14.555556	4.300783	1.013704	13.5	9	22	0.45607
		36	547	15.194444	5.052973	0.842162	14.0	4	26	0
Dumping site	Wet	38	105	2.763158	3.123011	0.506619	2.0	0	13	0.12011
	Dry	36	157	4.361111	5.259836	0.876639	3.0	0	24	0.12011
		74	262	3.540541	4.342177	0.504767	2.5	0	24	0
Abattoir site	Wet	52	139	2.673077	3.033933	0.420730	2.0	0	12	0.16547
	Dry	54	202	3.740741	4.638580	0.631230	3.0	0	23	0.16547
		106	341	3.216981	3.952105	0.383862	3.0	0	23	0

**FIGURE 2:**
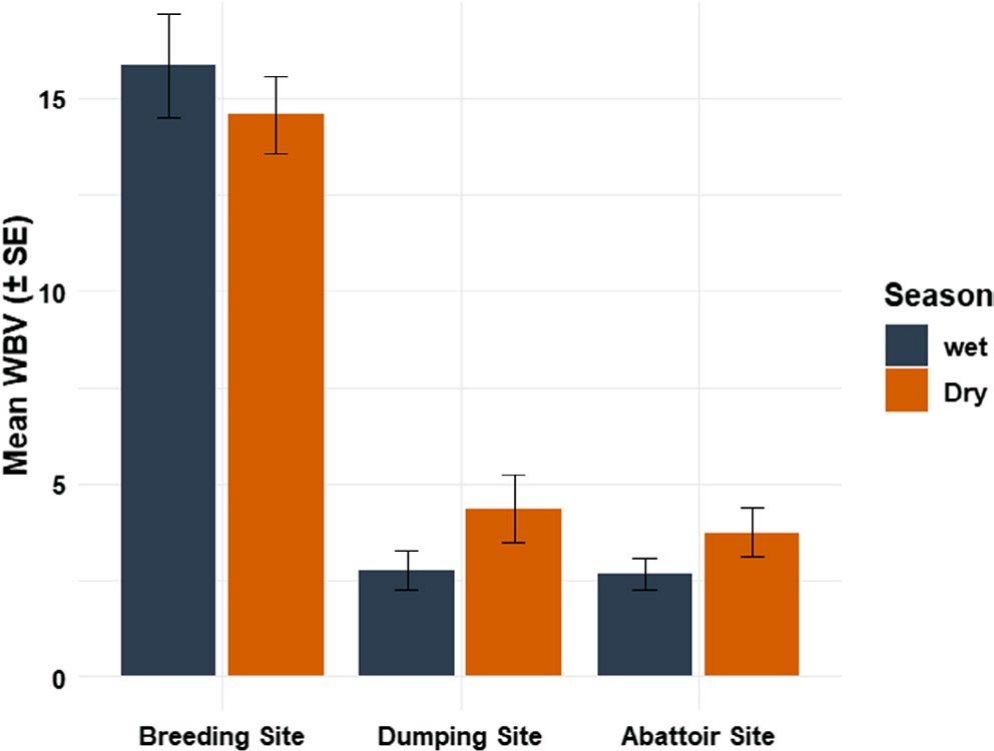
White‐backed vulture (WBV) population status in Southwest Ethiopia study sites by season. Breeding site (wet *N* = 285 and dry *N* = 262; total = 547), Dumping site (wet *N* = 105 and dry *N* = 157; total = 262), and Abattoir site (wet *N* = 139 and dry *N* = 202; total = 341).

The total number of vultures was slightly higher in the dry season (621 individuals) than in the wet season (529 individuals). The mean (± SD) count per observation was 5.75 ± 6.19 (median = 4.5) in the dry season and 4.90 ± 6.10 (median = 3.0) in the wet season. When stratified by site type, breeding sites showed a higher mean during the wet season (15.83 ± 5.76) than the dry season (14.56 ± 4.30), while dumping and abattoir sites both had higher means in the dry season (Figure 2). A two‐way ANOVA confirmed a strong effect of site type (*F*_2_,_210_ = 115.87, *p* < 0.0001, partial *η* ^2^ = 0.52) but no significant effect of season (*F*_1_,_210_ = 2.19, *p* = 0.141) nor interaction (*F*_2_,_210_ = 1.44, *p* = 0.239).

When observations were grouped into three time blocks (morning 7:00–12:00, early afternoon 12:00–16:00, late afternoon 16:00–18:00), the highest total count was recorded during the morning (*n* = 111, total = 536, mean ± SE = 4.83 ± 0.55) (Table 2). The late afternoon had the highest mean count (6.96 ± 1.16 SE) but fewer observations (*n* = 47, total = 327). Mid‐day recorded intermediate values (*n* = 58, total = 287, mean ± SE = 4.95 ± 0.63). A one‐way ANOVA indicated a significant effect of time block on white‐backed vulture counts (*F*_2_,_213_ = 3.98, *p* = 0.020). Tukey HSD post hoc tests revealed that the late afternoon mean was significantly higher than the morning mean (*p* = 0.029), while no other pairwise comparisons were significant (*p* > 0.05). When using the original “Diurnal Activity” categories (Morning vs. Afternoon), there was no significant difference (*t*_212.1_ = −1.241, *p* = 0.216).

**TABLE 2:** Diurnal activity patterns of white‐backed vultures in Southwest Ethiopia.

Time	*N*	Mean	SD	SE	Median	Min	Max	Total
Late afternoon (Garbett et al. 2018; Kendall and Bracebridge 2021; Murn and Anderson 2008)	47	6.95744	7.95628	1.16054	4	0	24	327
Early afternoon (Swan et al. 2006; Phipps, Wolter, et al. 2013; Phipps, Willis, et al. 2013; Green et al. 2004; Garbett et al. 2018)	58	4.94827	4.82108	0.6330	4	0	21	287
Morning (Gandaho et al. 2025; Murn et al. 2016; Virani et al. 2011; Buechley, Ruffo, et al. 2022; Ogada 2010; Swan et al. 2006)	111	4.82882	5.81983	0.55239	3	0	26	536

**FIGURE 3:**
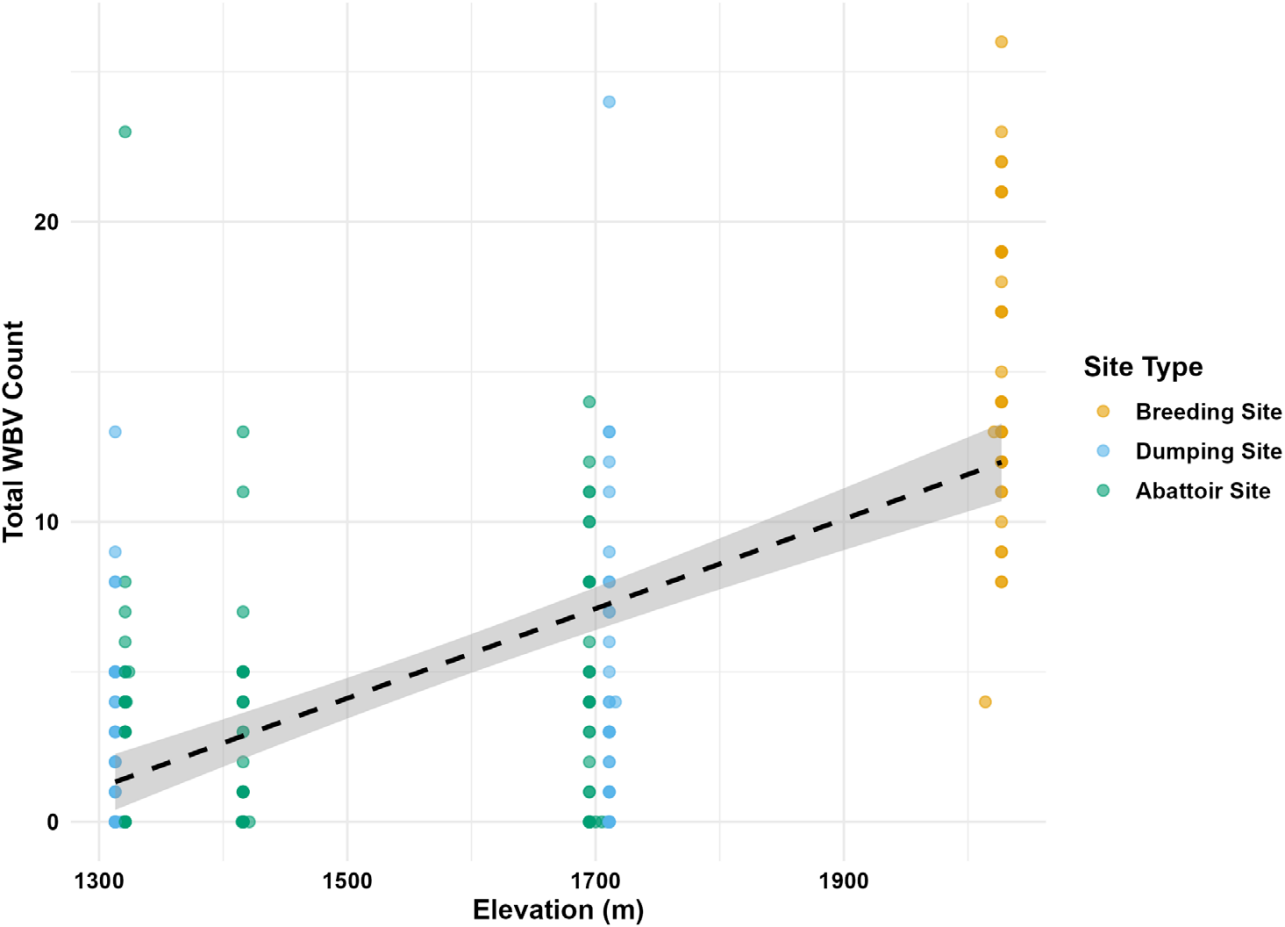
Observation of White‐backed Vulture (WBV) ( *Gyps africanus* ) counts relative to site elevation in southwestern Ethiopia, June 2024 to February 2025.

Breeding sites occurred at significantly higher elevations (mean ± SE: 2026.5 ± 0.39 m, range 2014–2027 m, *n* = 36) compared to dumping sites (1517.2 ± 23.2 m, range 1313–1716 m, *n* = 74) and abattoir sites (1473.4 ± 15.4 m, range 1321–1705 m, *n* = 106) (Figure 3). A one‐way ANOVA revealed a highly significant effect of site type on altitude (F_2_,_213_ = 166.6, *p* < 0.0001). Tukey HSD post hoc comparisons confirmed that breeding sites differed significantly from both dumping sites (mean difference = 509.3 m, 95% CI: 431.9–586.7, *p* < 0.0001) and abattoir sites (mean difference = 553.0 m, 95% CI: 479.6–626.5, *p* < 0.0001), while the latter two did not differ from each other (mean difference = −43.7 m, *p* = 0.176).

### 3.2 Age Structure Categories

**FIGURE 4:**
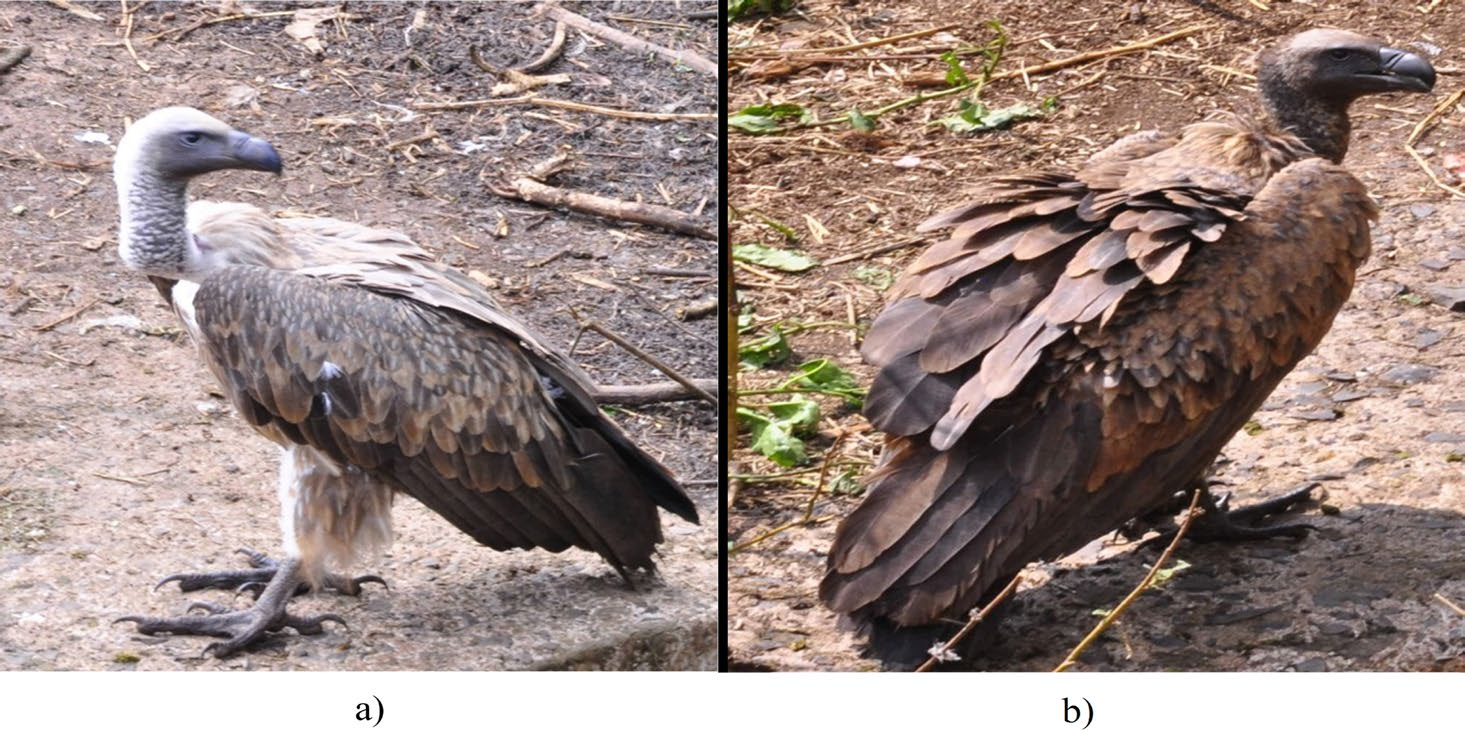
Age structure of white backed vulture (Photo by Asrat Aero Mamo, December 2024). (a) Adult: Characterized by predominantly dark plumage, a contrasting white rump and lower back (diagnostic in flight), white underwing coverts and a distinct white neck ruff. (b) Juvenile: Distinctive dark brown plumage with fine, light streaks and dark contour feathers; notably lacks the white neck ruff, white rump, and white underwing coverts characteristic of adults.

The age structure analysis revealed a population dominated by adult White‐backed vultures, which comprised 79.48% (*n* = 914) of all sightings while juveniles accounted for 20.52% (*n* = 236) of observations (Figure 4). The overall proportion of adults was 0.795, which deviated significantly from 0.5 (binomial test: *p* < 0.0001, 95% CI: 0.770–0.818). The age composition varied markedly by site type. Breeding sites had the highest juvenile proportion (27.1%; adult: juvenile ratio = 2.70:1), followed by dumping sites (17.6%; ratio = 4.70:1) and abattoir sites (12.3%; ratio = 7.12:1). A chi‐square test of independence revealed a strong association between site type and presence of juveniles (*χ* ^2^_2_ = 85.55, *p* < 0.0001). Juveniles were present in 91.7% of observations at breeding sites but only in 21.6% at dumping sites and 12.3% at abattoir sites. Conversely, there was no association between season and juvenile presence (*χ* ^2^_1_ = 0, *p* = 1.0). Levene's test indicated that variances were homogeneous across site types (*F*_2_,_213_ = 2.61, *p* = 0.076), supporting the use of parametric tests.

### 3.3 Habitat Preferences

During field observation, it was evident that vultures showed clear and repeated preferences for specific tree species across the Montane Forest landscape. The white‐backed vulture was often observed in larger, emergent trees that provided clear vantage points over the surrounding forest and open areas. A total of 906 tree observations were recorded across all study sites. Among the 13 tree species identified, the highest total white‐backed vultures counts were recorded on *Juniperus procera* (272 individuals, mean ± SE: 2.52 ± 0.28), *Olea welwetschia* (265: 2.45 ± 0.26), and *Podocarpus falcatus* (258: 2.39 ± 0.30). These three species also had the highest mean of African white‐backed vulture per observation (Table 3). All three species were used primarily for perching (used during the day for scanning, resting and feeding‐associated activities) and roosting (used overnight and during prolonged resting periods), with occasional signs of nesting activity noted in the largest individuals (e.g., stick platforms in *J. procera* and *P. falcatus* ). The lowest total counts were found on *Cordia africana* (120, mean ± SE: 1.11 ± 0.11) and *Prunus africana* (91, mean ± SE: 0.84 ± 0.13). A chi‐square test of independence on the top 10 tree species (those with highest total of white‐backed vulture) revealed a highly significant association between tree species and African white‐backed vulture presence (*χ* ^2^_9_ = 21.23, *p* = 0.0117), confirming that African white‐backed vulture do not use all tree species equally.

**TABLE 3:** Tree Species Preferences of white‐backed vultures ( *Gyps africanus* ) in study sites.

Scientific name	Common name	N with vulture	Total	Mean	SD	SE
*Juniperus procera*	African juniper	72	272	2.518519	2.882371	0.277356
*Olea welwetschia*	Elgon olive	85	265	2.453704	2.673456	0.257253
*Podocarpus falcatus*	Outeniqua yellowwood	73	258	2.388889	3.09003	0.297338
*Juniperus procera*	African pencil‐cedar	81	222	2.055556	1.898516	0.182685
*Croton macrostachyus*	Rushfoil	81	220	2.037037	1.701708	0.163747
*Polyscias fulva*	Parasol tree	73	213	1.972222	2.357959	0.226895
*Allophyllas abyssinicum*	Forest velvet false‐currant	67	177	1.638889	1.676993	0.161369
*Ficus vasta*	Ethiopian fig	64	167	1.560748	2.249434	0.216452
*Syzigium guineense*	Waterberry or Water‐pear	74	163	1.509259	1.53157	0.147375
*Albizia gumifera*	Peacock flower	62	151	1.398148	1.734422	0.166895
*Sapium ellipticum*	Jumping seed tree	69	149	1.37963	1.392353	0.133979
*Cordia africana*	Sudan teak	64	120	1.111111	1.194484	0.114939
*Prunus Africana*	African cherry	44	91	0.842593	1.33369	0.128334

Breeding sites contributed the fewest tree observations (*n* = 234) but still accounted for a substantial total of 470 white‐backed vulture (mean ± SD: 2.01 ± 2.41). Dumping sites had 468 observations (749 white‐backed vulture, mean ± SD: 1.60 ± 1.83) and abattoir sites 702 observations (1249 white‐backed vulture, mean ± SD: 1.78 ± 2.18). A one‐way ANOVA indicated a marginally significant effect of site type on African white‐backed vulture counts (*F*_2_,_1400_ = 2.99, *p* = 0.0508). Tukey HSD post hoc tests showed that dumping sites had significantly lower white‐backed vulture counts than breeding sites (difference = −0.408, 95% CI: −0.805 to −0.011, *p* = 0.042). No significant differences were found between abattoir and breeding sites (*p* = 0.330) or between abattoir and dumping sites (*p* = 0.322). A chi‐square test for site type versus white‐backed vulture presence was not significant (*χ* ^2^_2_ = 2.32, *p* = 0.314), suggesting that while counts differ, the proportion of trees used does not vary strongly among site types.

## 4 Discussion

The population status and habitat preferences of African White‐backed vultures in southwest Ethiopia's montane forests reveal both unique regional patterns and striking parallels with global vulture ecology.

### 4.1 Population Status

The overall mean count of 5.32 African white‐backed vulture per observation recorded in this study falls within the range reported from other African savanna ecosystems. However, the striking disparity in abundance among the three site types, with breeding sites harboring 15.19 ± 5.05 individuals per observation compared to 3.54 and 3.22 at dumping and abattoir sites, respectively, highlights the critical role of undisturbed breeding habitats. These findings align with studies from elsewhere in Africa and other continents. Slaughterhouses (abattoirs) act as powerful attractants for vultures due to the reliable daily waste (Agunbiade et al. 2025). A study in Ethiopia found that vultures contributed 57% of carrion removal at these sites (Buechley, Ruffo, et al. 2022). The strong pull of this resource is a global phenomenon, with one study in Spain recording that over 62% of observed feeding events for Eurasian Griffon Vultures occurred at predictable sites like carcass dumping grounds and intensive farms (Fernández‐Gómez et al. 2022). The same study in Wolkite town, Southwestern Ethiopia, found that 472 individuals (36%) were counted at dumping sites (mean ± SD: 20 ± 7.4) and 839 individuals (64%) at abattoirs (mean ± SD: 34 ± 8), further confirming the importance of both site types for white‐backed vulture populations in Ethiopia (Shiferaw et al. 2022). The present study extends these findings by demonstrating that breeding sites not only serve as nesting locations but also support substantially higher overall population size compared to foraging sites (abattoirs and dumps) (Gebrekidan and Bogale 2021).

The significant diurnal variation in African white‐backed vulture ( *Gyps africanus* ) counts (ANOVA: *F*_2_,_213_ = 3.98, *p* = 0.020), with late afternoon counts significantly higher than morning counts (*p* = 0.029), reflects behavioral patterns observed in other vulture species. Studies of White‐rumped Vultures ( *Gyps bengalensis* ) in Nepal found that perching occurred most frequently in the late afternoon, accounting for 36% of observations (Koirala et al. 2023). This behavior likely relates to thermoregulation, as vultures use thermal updrafts that develop as the day warms, with late afternoon representing a peak activity period before roosting.

No significant difference when using only two categories (morning vs. afternoon) underscores the value of the three‐block classification adopted in this study. The intermediate values recorded during mid‐day (mean ± SD: 4.95 ± 0.63, *n* = 58) suggest that white‐backed vulture activity is relatively consistent throughout daylight hours, with a distinct peak in the late afternoon that would have been masked by a simple morning‐afternoon dichotomy. A study by Kendall (2014), which examined temporal segregation among vulture species, found that White‐backed Vultures were consistently more abundant at carcasses in the morning than in the afternoon throughout the year (Kendall 2014).

Dumping and abattoir sites showed higher counts in the dry season than in the wet season, while breeding sites showed the opposite pattern. The trend toward higher dry season usage of dumping and abattoir sites is consistent with the hypothesis that natural carrion becomes scarcer during the dry season, forcing vultures to rely more heavily on anthropogenic subsidies (Fernández‐Gómez et al. 2022). In many African savannas, wild ungulate mortality peaks in the late dry season, but carcasses are rapidly consumed or desiccated, making them less available over time (Moleón et al. 2014).

Breeding sites occurred at significantly higher elevations than dumping and abattoir sites of southwest Ethiopia region, suggesting that elevation is a strong predictor of white‐backed vulture habitat selection. This high‐elevation breeding sites serve as refugia from human pressure and its associated risks, such as infrastructure, habitat conversion and direct disturbance. For Rüppell's vultures ( *Gyps rueppellii* ) in Kenya, precipitation and elevation emerged as the primary environmental predictors of distribution (Chepkirui et al. 2024). This was closely related to *Gyps* species; elevation plays a very similar role in defining suitable habitat. Similarly, Egyptian vultures in central‐west Nepal preferred heterogeneous habitats at high altitudes, which may be relatively difficult for humans to access (Gurung et al. 2022). Also, elevation here serves as a surrogate for landscape type; high‐elevation breeding sites are located in Afromontane forests, where human disturbance is lower (Shiferaw et al. 2022). In contrast, low‐elevation dumping and abattoir sites are situated near towns and agricultural areas, where livestock waste dominates. This spatial segregation reinforces the idea that vultures must commute between high‐elevation breeding/roosting habitats and low‐elevation foraging sites (Bamford et al. 2008).

### 4.2 Age Structure

The age structure analysis revealed a population heavily dominated by adults (79.48%, *n* = 914), a pattern consistent with other critically endangered vulture populations. The adult‐skewed age ratio in White‐backed vulture populations raises significant conservation flags when compared to benchmark populations in South Africa (70%–75%) (Phipps, Willis, et al. 2013), Zambia (68%–72% adults) (Murn and Anderson 2008) and Kenya (65%–70% adults) (Monadjem et al. 2012). This suggests potential reproductive challenges including low fledging success due to food limitation, high juvenile mortality from anthropogenic threats, and delayed breeding age from environmental stressors. The finding that adult white‐backed vulture constitutes the vast majority of the population is not unique to this study but appears to be a consistent demographic trait across the *Gyps* genus. Studies of Long‐billed ( *Gyps indicus* ) and White‐rumped ( *Gyps bengalensis* ) vultures in India found that adults constituted 78% and 80% of populations, respectively, with low proportions of juveniles and sub‐adults (Ravikanth and Baskaran 2024). This is also evident in European populations of the Griffon Vulture ( *Gyps fulvus* ), a close relative. A study on the island of Crete found that adult birds comprised 63% of the estimated total population of 379 individuals (Xirouchakis and Mylonas 2005). Research in Portugal further demonstrates that the Griffon Vulture population is most sensitive to juvenile mortality, with a decrease in food supply leading to a decline in vulture abundance and negatively affecting the spatial distribution of the species by reducing the number of breeding pairs per colony. This highlights the crucial role of recruitment and the vulnerability of these slow‐reproducing species (van Beest et al. 2008).

The pronounced site‐specific variation in age structure observed in this study where juveniles were almost exclusively found at breeding sites while being conspicuously absent from dumping and abattoir sites. The strong association between site type and juvenile presence appears to be a relatively common feature of African vulture ecology (Kendall and Bracebridge 2021; Ravikanth and Baskaran 2024). The concentration of juveniles at breeding sites is likely driven by several reinforcing factors (parental dependence; there may be a conservative dispersal strategy where inexperienced young birds may not venture far from familiar breeding grounds, and adult dominance could play a role) (Ravikanth and Baskaran 2024).

### 4.3 Habitat Preferences

The habitat preference results of the present study provide important insight into the roosting ecology of White‐backed Vultures ( *Gyps africanus* ) in the Ethiopian highlands. The clear and strong selection for *Juniperus procera*, *Olea welwetschia*, and *Podocarpus falcatus* confirms the global pattern that this species requires tall, structurally sound trees for roosting, sunning, and scanning surrounding areas while also highlighting a unique local adaptation to Afromontane forest species that sets this population apart from others across sub‐Saharan Africa (Gandaho et al. 2025; Tom 2025). A continental analysis identified Ethiopia as a stronghold for vulture conservation in Africa; indeed, one‐sixth of the core distribution of the White‐backed Vulture was located in Ethiopia, validating the national and global importance of this population (Buechley, Ruffo, et al. 2022).

The analysis of tree species preferences revealed that *Juniperus procera* (native) (272 individuals, mean ± SE: 2.52 ± 0.28), *Olea welwetschia* (native) (265, mean ± SE: 2.45 ± 0.26), and *Podocarpus falcatus* (native) (258, mean ± SE: 2.39 ± 0.30) were the most heavily utilized tree species. *Juniperus procera* recorded the highest total white‐backed vulture count of any tree species in southwest Ethiopia (Teklemariam and Verma 2013). This native conifer is a dominant element of the Afromontane flora in eastern Africa, capable of reaching 20–25 m in height and in its natural habitat it can attain heights of up to 60 m and live for hundreds of years (Zenebe et al. 2024). The large trunk diameter and dense, widespread crown of mature *J. procera* provide a stable, well‐elevated platform for nesting, roosting and its structural robustness likely reduces the risk of nest collapse, a factor that is likely to be important for a species that builds a large stick nest and uses the same nest site repeatedly over many years (Ruby Yadav and Kanaujia 2023). White‐backed vultures in South Africa preferentially nest in larger trees, especially *Diospyros mespiliformis* , with tree size being a stronger predictor of nest presence than tree health. In the Associated Private Nature Reserves of South Africa, vultures utilized 10 tree species, favoring the largest, least impacted trees with greater stability and longevity (Gandaho et al. 2025). *Podocarpus falcatus* is one of the largest native trees in Ethiopia, reaching heights of up to 45 m and a diameter of 250 cm in its natural environment. Its natural range extends from 1500 to 2500 m in the central and eastern highlands, covering exactly the elevation of the breeding sites studied. The large, dense crown and its position above the forest canopy make it an ideal observation post, allowing vultures to scan for carrion over long distances (Hounnouvi et al. 2025). *Olea welwetschia* is a low‐to‐tall evergreen tree attaining heights of 12–24 m, with a native range restricted to Ethiopia and further south in tropical Africa. It is a member of the characteristic Afromontane rainforest tree community, along with *Syzygium guineense*, *Polyscias fulva*, *Diospyros abyssinica* and *Cordia africana*. As a tall, naturally occurring tree within the breeding elevation zone, it provides a similarly large and stable nesting platform. The fact that white‐backed vultures utilize this native species is consistent with the broader pattern observed across Africa: vultures select the largest, most stable trees available to them (Gandaho et al. 2025; Laizer et al. 2026).

### 4.4 Conservation Implication

Based on the findings of this study, several conservation implications emerge for the African White‐backed Vulture in Southwest Ethiopia region. Breeding sites, which harbor the highest vulture numbers and are the only areas where juveniles are regularly recorded (present in 91.7% of observations), depend entirely on large, mature trees of three native Afromontane species: *Juniperus procera, Olea welwetschia*, *and Podocarpus falcatus*. The legal protection of these trees, together with the establishment of buffer zones around active colonies to limit road construction, logging, and agricultural expansion, is the highest priority. In contrast, abattoirs and dumping sites attract mainly adults and provide supplementary food, especially in the dry season, but also expose vultures to poisoning, competition with feral dogs, and other anthropogenic threats. To mitigate these risks, we recommend creating Vulture Safe Feeding Sites away from existing facilities, banning the use of toxic NSAIDs in livestock, and initiating a long‐term monitoring programme that tracks vulture numbers, nesting tree condition, and poisoning incidents. Without immediate implementation of these measures, Ethiopia risks losing one of its last strongholds for this Critically Endangered species.

### 4.5 Conclusion and Recommendations

This study confirms that southwest Ethiopia's Montane Forests are home to the African white‐backed vulture, with unique yet interrelated conservation needs. The results highlight the crucial necessity of protecting breeding cliffs and key tree species, particularly *Juniperus procera, Olea welwetschia*, *and Podocarpus falcatus*, which serve as essential habitat features. Additionally, there is a significant need for targeted interventions at anthropogenic sites to reduce the risk of poisoning, as well as urgent action to address apparent reproductive challenges indicated by skewed age ratios. Finally, while it is valuable to draw from conservation experiences in other regions, it remains imperative to develop strategies tailored specifically to the Ethiopian context.

We recommend that local authorities consider establishing buffer zones around known nesting colonies to restrict new infrastructure, logging and intensive agriculture. Additionally, implementing strictly managed supplementary feeding (e.g., Vulture Safe Feeding Sites away from abattoirs, using NSAID‐free meat with veterinary oversight) to provide a clean, predictable food source and reduce poisoning and disease risks.

These measures, combined with community engagement and enforcement of wildlife protection laws, offer the best hope for preserving these ecologically vital scavengers in Ethiopia's changing landscapes. The study provides a crucial baseline for future monitoring and represents an important contribution to global vulture conservation efforts, particularly for understudied montane forest populations. Continued research should focus on reproductive success rates, movement ecology, and the specific threats contributing to the observed age structure imbalances.

## Author Contributions

**Asrat Aero Mamo:** conceptualization (supporting), data curation (lead), formal analysis (lead), funding acquisition (lead), investigation (equal), methodology (lead), project administration (lead), resources (lead), writing – original draft (lead). **Tsegaye Gadisa:** conceptualization (supporting), data curation (supporting), funding acquisition (supporting), investigation (supporting), methodology (supporting), supervision (lead), writing – original draft (supporting), writing – review and editing (supporting). **Tadesse Habtamu:** conceptualization (supporting), data curation (supporting), funding acquisition (supporting), methodology (supporting), supervision (lead).

## Funding

This work was supported by the Bonga University and Jimma University, with additional material support from Prof. Nubia Kai and Mr. Teketel Bafa as a personal and also material grant from Idea Wild.

## Conflicts of Interest

The authors declare no conflicts of interest.

## Data Availability

The data supporting the findings of this study, including all analyzed figures, are openly available in figshare at the following https://doi.org/10.6084/m9.figshare.31169362.
